# Isabella Barbour ‘Ella’ Pirrie (1857–1929): Pioneering contributions to British nursing

**DOI:** 10.1177/09677720241273661

**Published:** 2024-08-18

**Authors:** Hareesha Rishab Bharadwaj, Muhammad Hamza Shah, Kahan Mehta

**Affiliations:** 1 Faculty of Biology Medicine and Health, 5292 The University of Manchester, Manchester, UK; 2 School of Medicine, Dentistry & Biomedical Sciences, 1596 Queen's University Belfast, Belfast, UK; 3 Department of Medicine, GMERS Medical College, Vadodara, India

**Keywords:** Nursing, mentorship, UK

## Abstract

Miss Isabella “Ella” Barbour Pirrie (1857–1929) made substantial contributions to nursing through her work in Belfast and Edinburgh. Born to a notable medical family, Pirrie's inclination toward nursing was influenced by her father's profession. She trained at the Liverpool Royal Infirmary and was mentored by Florence Nightingale, whose guidance shaped her nursing philosophy and practices. Notably, Pirrie's role in the Belfast Union Workhouse Infirmary was transformative; she championed the implementation of standardized nursing uniforms, enhancing the professional standing and recognition of nurses. Her efforts led to the establishment of a formal nursing training program in Belfast, despite facing significant resistance and challenges. In 1894, Pirrie moved to Edinburgh to become the First Matron at the Lady Grisell Baillie Memorial Hospital. Her tenure there was marked by significant advancements in nursing education, including the establishment of a community and district nursing department. By the end of her service, over 140 nurses had been trained, with many pursuing international missions. Despite her resignation in 1914 due to health issues, Pirrie continued her work as the superintendent of the Deaconess Rest Home in Edinburgh until her death in 1929. Her legacy is commemorated by a statue at Belfast City Hospital and a plaque at Greyfriars' Charteris Sanctuary, reflecting her profound impact on nursing education and the professionalization of the field.

Miss Isabella “Ella” Barbour Pirrie, DCS (Deaconess Church in the Scotland), was born on June 5, 1857, as the third of twelve children to Dr John Miller Pirrie (1824–1873) and Isabella Barbour (1827–1873).^1^

Her father, Dr John Miller Pirrie, was a notable medical practitioner affiliated with the Belfast Union Infirmary and the Royal Hospital in Belfast. He also served as president of the Belfast Medical Society from 1858 to 1859.^1,2^ Her mother, Isabella Barbour, was descended from John Barbour, (1765–1823) a key figure in the linen trade near Lisburn, Northern Ireland.^1,3^ Isabella's extended family included prominent figures such as her first cousin, shipbuilder William James Pirrie, 1st Viscount Pirrie (1847–1924), and her second cousin, John Miller Andrews (1871–1956), the second prime minister of Northern Ireland.^4^

The exact catalyst for her inclination towards nursing as her vocation remains somewhat enigmatic; however, it is widely speculated that this choice was shaped by her father's occupation. Consequently, Miss Pirrie commenced formal training at the Liverpool Royal Infirmary, situated in Pembroke Place in Liverpool, England, and thereafter engaged in collaborative work with Mr Edward Robert Bickersteth (1828–1908), a resident surgeon within the institution.^5^

## Nurturing bonds of mentorship: Florence nightingale's influence on Isabella Pirrie's nursing journey

Although Ella Pirrie was not enrolled as a student at Florence Nightingale's Training School for Nurses at St Thomas’ Hospital, she greatly benefited from Nightingale's guidance and mentorship. Throughout many years, Nightingale maintained a close connection with Pirrie, exchanging numerous letters with her while she resided in Belfast. Despite coming from different generations, both women hailed from affluent British families and shared respect for the nursing profession, firmly believing it was their calling.^1,6,7^ This commonality fostered a strong bond between them.

Pirrie's relationship with Nightingale, though informal, was instrumental in her growth and development as a nurse. The regular correspondence between them provided Pirrie with invaluable insights and advice, shaping her nursing philosophy and practices. Nightingale's emphasis on sanitation, patient care, and the professionalisation of nursing likely had a profound impact on Pirrie. This connection also extended to practical advice on career development and training opportunities.^7^ It is possible that Nightingale's influence led Pirrie to seek further training with the local Deaconesses in Berlin, Germany, as Nightingale herself had done. Nightingale was known for advocating for the Deaconess training system, which emphasised rigorous nursing education and moral development. Pirrie's decision to train in Berlin could have been inspired by Nightingale's experiences and recommendations, illustrating the depth of Nightingale's impact on her career.

Pirrie returned to Belfast in 1884 after her training in Berlin, though the details of her time there remain unclear.^8^ This period of advanced training abroad likely enhanced her skills and broadened her nursing perspective, further contributing to her effectiveness and influence in the nursing profession. Nightingale's mentorship thus played a crucial role in Pirrie's professional journey, underscoring the enduring impact of their relationship on Pirrie's contributions to nursing.

## Elevating nursing through advocacy

In November 1884, Miss Pirrie was appointed Superintendent and Head Nurse at the Belfast Union Workhouse Infirmary (now the Belfast City Hospital), where her father had previously served as a physician, with a yearly salary of £30.^9^ The nursing facilities at Belfast City Hospital at the time had been operating in a poor and unorganised state that resulted in a substandard quality of care. Until the 1880s, the nursing work at the institution had been carried out entirely by untrained women, with most of the handy work being carried out by paupers.^1,9^ Furthermore, the lack of standardised uniforms among nurses was a prevalent issue that had significant implications for patient care and the nursing profession as a whole.^1^ The nurses in the past often wore ordinary clothing, which made it difficult for patients to identify as healthcare providers. This lack of distinction leads to confusion and delays in receiving essential medical attention. Without standardised uniforms, the professional image and identity of nurses were not as pronounced, affecting the level of recognition they received for their crucial role in patient care.^1,9^

Isabella Pirrie played a pivotal and transformative role in championing the implementation of standardised uniforms for paid nurses, a significant stride towards elevating nursing's professional standing within healthcare, as evidenced by historical records.^9^ Her approach was marked by persistent advocacy, driven by a profound desire to secure greater recognition and esteem for the nursing vocation. Despite lacking formal training, the nurses serving at the Belfast Union Workhouse Infirmary played indispensable roles, yet their invaluable contributions remained unacknowledged, often accompanied by disdainful treatment.^9^ By championing the implementation of uniforms, Pirrie sought to establish a formal recognition of nursing's indispensable role within the institution, signifying a pivotal initial stride toward securing the rightful reverence and acknowledgment the profession merited.^1,9^ After a month of dedicated advocacy, the institution's guardians ultimately sanctioned uniforms for the paid nurses, accompanied by distinctive aprons for the unpaid female attendants. Nevertheless, progress was sluggish, frequent vacancies among the untrained nurses coupled with the guardians’ reluctance to incur additional expenses posed hurdles to rapid advancement.^1^

## Nurturing transformation: Isabella Pirrie's vision for nursing training

In October 1885, the first formal meeting took place between Pirrie and Nightingale in London. During this meeting, Pirrie apprised Nightingale of the transformative changes she had advocated for at the Belfast Union Workhouse Infirmary. As a result, Pirrie's stature in Nightingale's eyes was further heightened. Subsequent to this encounter, Florence Nightingale is said to have dispatched numerous letters filled with words of encouragement to Pirrie, solidifying their bond.^1,10^

While in London, Pirrie engaged in further training, culminating in her attainment of a diploma from the London Obstetrical Society in October 1886.^1^ Subsequently, upon her return to Belfast, she resumed her position at the Belfast Workhouse Infirmary. Inspired by Nightingale's counsel, Pirrie embarked on advocating for the establishment of formal nursing training within the institution.^1,10^ The prevailing absence of comprehensive training for nurses at the infirmary constituted a pervasive issue with wide-ranging repercussions for patient care. Furthermore, the absence of formal training perpetuated a negative perception of the nursing profession, relegating it to a status that seemed undeserving of systematic education and preparation.

In lieu of this, Pirrie began a determined campaign to establish a formal nursing training programme within the institution. However, she faced significant resistance and numerous challenges. Convincing the Belfast Guardians to consider her proposal was an arduous task, compounded by the prevailing gender biases of the era that often dismissed women's professional insights. Additionally, there was vehement opposition from the law commissioners in Dublin, who were sceptical about the feasibility and necessity of such a programme.^1^ Despite these obstacles, a sub-committee of the Belfast Guardians was convened in early 1887 to deliberate on the feasibility of introducing nursing training at the Belfast Union Infirmary. Following careful discussions, by 1888, the guardians accepted Pirrie's proposal to initiate the training of six suitable women, aged in their twenties to early thirties, as probationers. This comprehensive three-year training programme included a salary of £10 for the first year, £15 for the second year, and £18 for the third year.^1,11^ Upon successful completion of their training, these individuals would join the ranks of the paid nursing staff.

## The move to Edinburgh

In September 1891, Isabella Pirrie submitted her resignation from the Belfast Union Workhouse Infirmary, citing overwhelming stress and exhaustion as the primary reasons.^5^ However, after a month-long hiatus, she resumed her position. The institution's management, overseen by the guardians, believed Pirrie's presence was essential. In an interview conducted by the Northern Whig in 1891, the management stated, ‘The benefits accruing to the Infirmary by Miss Pirrie's excellent management are scarcely to be described by mere words’.

In 1894, an opportunity arose for the position of First Matron at the newly established Lady Grisell Baillie Memorial Hospital, affiliated with the Church of Scotland Deaconess Hospital in Edinburgh.^1,5^ The vacancy was not publicly advertised, rather, the institution actively approached Isabella Pirrie for the role. The hospital aimed to offer specialized training in nursing for women aspiring to participate in home mission activities. Miss Pirrie, a woman of strong religious conviction, regularly offered her services as deaconess-superintendent to the Church of Scotland without a salary. Consequently, she decided to leave Belfast for Edinburgh in the same year.^1,4,5^

Throughout her service at the institution, Pirrie played a pivotal role in advancing nursing education. After completing her inaugural year as the First Matron, she was invited to join the hospital's board of governors, underscoring her significance and influence.^1^ She was instrumental in the growth and enhancement of the in-hospital nursing division and led the establishment of a community nursing and district nursing department, both under her supervision.^5^ By the end of 1914, over 140 nurses had successfully completed the training programme, with more than 90 embarking on international missions.^1,5^ However, the years of dedicated nursing and administrative responsibilities took a toll on her physical health and overall well-being. In May 1914, at the age of 56, Miss Pirrie tendered her resignation from the institution, despite the hospital administration's reluctance to accept her decision.^5^ Nonetheless, her commitment to nursing remained steadfast. She subsequently assumed the position of superintendent at the Deaconess Rest Home in Edinburgh, where she continued her work until her death in 1929.^1,4^

## Legacy

Throughout her years in the nursing profession, Miss Isabella Pirrie championed many initiatives, significantly enhancing the recognition and respect for nursing. Her dedication led to the comprehensive training of numerous nurses, preparing them for service both in the United Kingdom and abroad. Her efforts did not go unnoticed. In 1910, Sir Edward Coey Bigger (1861–1942), the medical inspector of the Local Government Board, praised her work at the Belfast Union Infirmary, stating,

I have never known in connection with an institution any more self-sacrificing and noble work than that performed by Miss Pirrie. It would be impossible to overestimate the lasting benefits to the sick conferred by the change in the nursing system which commenced in 1884.^1^

**Figure 1.**
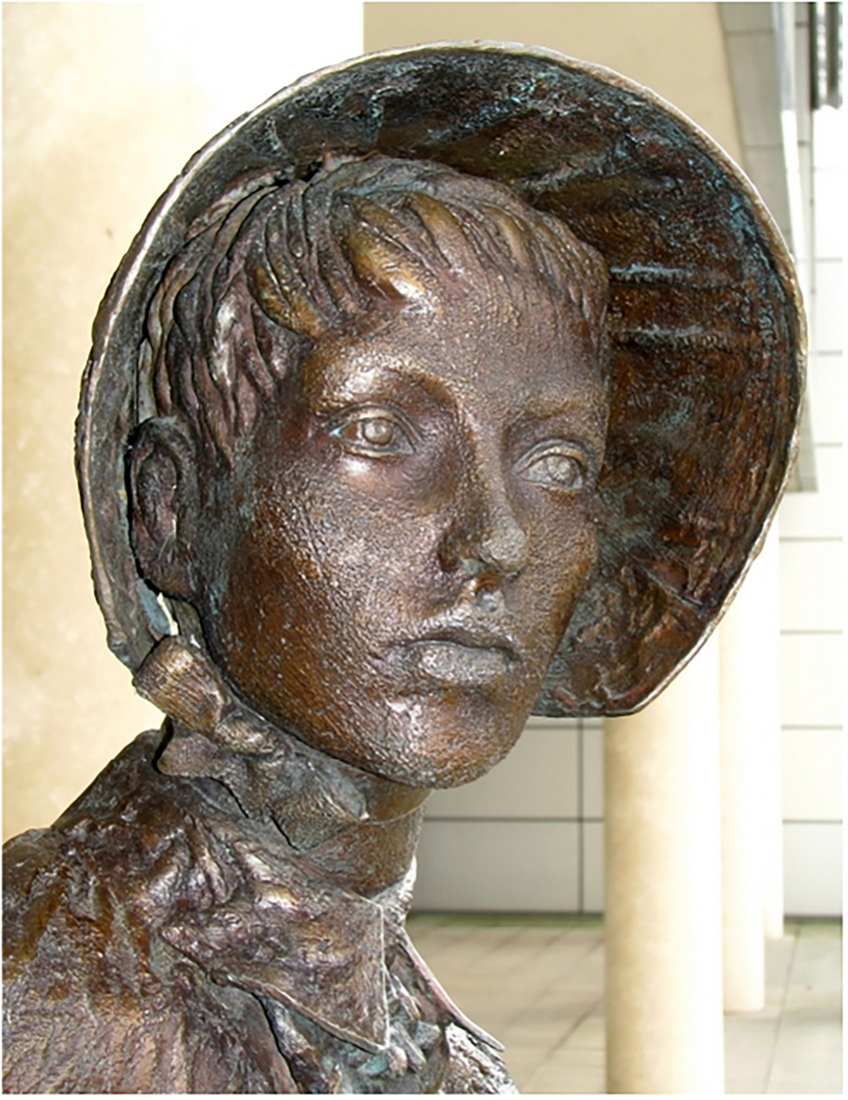
The statue of Isabella Pirrie at the Belfast City Hospital, Belfast, Northern Ireland.^12^.

This statue of Isabella Barbour Pirrie, crafted by the artist Ross Wilson and prominently displayed at the Belfast City Hospital, stands as a profound testament to her resolute and unwavering commitment to the noble nursing profession (Figure 1). The statue of Pirrie at Belfast City Hospital illustrates her holding one of the letters from Florence Nightingale, sent from London on 14 October 1885, that reads:

How deep my interest, how intense my feeling for you and your work. Every woman must feel the same. You have done a noble deed, God will grant you success. You have already done great things. You must be the nucleus of hope for a goodly future of trained nursing staff at Belfast Infirmary which needs you and of perhaps a future training school for Nurses. Godspeed to you. I am always saying in my heart. God bless you and your work always.^1,10^

In addition, a plaque, located at the Greyfriars’ Charteris Sanctuary in Edinburgh, designed to immortalise her efforts, reads:

To the glory of God and in grateful and loving memory of Ella Pirrie, Deaconess who rendered valuable aid in the equipment of the Deaconess Hospital of which she was the first matron from 1894 to 1914. She was the Superintendent of the Deaconess Rest House, Edinburgh from 1916 to 1923. Entered into rest 30th October 1929. ‘A Succourer of many.’ Erected by her fellow workers and other friends.^13^

## References

[1] Graffin S. Pirrie, Isabella Barbour [Ella] (1857–1929), nurse and nurse educator. [cited 2021 Apr 8]. Available from: https://www.oxforddnb.com/display/10.1093/odnb/9780198614128.001.0001/odnb-9780198614128-e-90000380678?rskey=DkuO9I&result=1.

[2] *Ulster Medical Society: Royal Victoria Hospital [Internet]* . www.ums.ac.uk. [cited 2023 Aug 10]. Available from: https://www.ums.ac.uk/rvh.html.

[3] *Barbour, John. Dictionary of Irish Biography [Internet]* . www.dib.ie. [cited 2023 Aug 10]. Available from: https://www.dib.ie/biography/barbour-john-a0364.

[4] *Ella Pirrie: Nurse (1857-1929)* . Biography, Facts, Information, Career, Wiki, Life [Internet]. peoplepill.com. [cited 2023 Aug 10]. Available from: https://peoplepill.com/people/ella-pirrie/tc/sports/.

[5] Macrae H . University of California Libraries. Alice Maxwell, deaconess [Internet]. Internet Archive. London: Hodder and Stoughton, 1919, [cited 2023 Aug 10]. Available from: https://archive.org/details/alicemaxwelldeac00macr/page/n15/mode/2up.

[6] *History.com Editors* . Florence Nightingale [Internet]. HISTORY. A&E Television Networks; 2009. Available from: https://www.history.com/topics/womens-history/florence-nightingale-1.

[7] Nightingale F Mcdonald L . Florence Nightingale: extending nursing. Waterloo, On: Wilfrid Laurier University Press, 2009.

[8] McNeill E Wright D Demetriades A . A brief look at the history of the deaconess hospital, Edinburgh, 1894–1990. Journal of the Royal College of Physicians of Edinburgh 2018; 48: 78–84. 29741534 10.4997/JRCPE.2018.118

[9] Craig D . A HISTORY OF THE BELFAST CITY HOSPITAL [Internet]. [cited 2023 Aug 10]. Available from: https://www.ums.ac.uk/inst/hbch_dc.pdf.

[10] McDonald L . ed. The collected works of Florence Nightingale, vol. 13: Florence Nightingale—extending nursing. 2009;pp. 694–8.

[11] O’Sullivan JF . Belfast City Hospital: a photographic history [Internet]. Library Catalog (Koha). Donaghadee: Ballyhay Books; 2003 [cited 2023 Aug 11]. Available from: https://library-search.nics.gov.uk/cgi-bin/koha/opac-detail.pl?biblionumber=68433.

[12] *Wikimedia Commons* . Ella Pirrie Statue, Belfast City Hospital [Internet]. Wikimedia Commons. [cited 2024 Jul 12]. Available from: https://commons.wikimedia.org/wiki/File:Ella_Pirrie_statue,_Belfast_City_Hospital_(detail)_-_geograph.org.uk_-_939603.jpg.

[13] *Women of Scotland* . Mapping Memorials to Women in Scotland - Plaque to Ella Pirrie [Internet]. Women of Scotland. [cited 2023 Aug 9]. Available from: http://womenofscotland.org.uk/memorials/plaque-ella-pirrie.

